# MRI-based evidence of rectus capitis posterior minor hypertrophy and its correlation with disease duration in Parkinson’s disease

**DOI:** 10.1186/s12880-026-02571-1

**Published:** 2026-07-08

**Authors:** Xin-Ting Liu, Cong Liu, Xin Fang, Chen-Xu Zhao, Sheng-Bo Yu, Jian-Fei Zhang, Nan Zheng, Qiang Xu, Hong-Jin Sui

**Affiliations:** 1 Department of Anatomy, Dalian Medical University, Dalian, 116044 China; 2 Department of Radiology, The 967th Hospital of the Joint Logistics Support Force of PLA, Dalian, 116021 China; 3 Department of Radiology, The First Affiliated Hospital of Dalian Medical University, Dalian, 116021 China

**Keywords:** Parkinson’s disease, Myodural bridge complex, Rectus capitis posterior minor, Cerebrospinal fluid, Occipito-atlantal cistern, Biomechanical coupling

## Abstract

**Background:**

Recent research in Parkinson’s disease (PD) has increasingly focused on alterations in cerebrospinal fluid (CSF) dynamics and glymphatic system (GS) function. The myodural bridge (MDB), a structure connecting the suboccipital muscles to the spinal dura mater, is involved in the regulation of CSF dynamics. The rectus capitis posterior minor (RCPmi) is a core component of the myodural bridge complex (MDBC). This study employed magnetic resonance imaging (MRI) to evaluate morphological changes in the RCPmi in PD patients and to explore their potential association with CSF dynamics and disease duration.

**Materials and methods:**

This retrospective MRI study included 349 participants: 156 patients with PD and 193 healthy controls (HC). The occiput-C2 angle (OC2A) was measured on mid-sagittal T2-weighted images. Standardized cross-sectional area (CSA) ratios of MDBC constituent muscles (RCPmi, RCPma, OCI) and non-constituent muscles (OCS, RCL) were quantified on axial images. In 51 PD patients with complete disease duration records (range 1–14 years), the correlation between RCPmi CSA and disease duration was analyzed. From the overall cohort, 277 participants with well-defined occipito-atlantal cistern (OAC) morphology (114 PD, 163 HC) were selected for measurement of OAC parameters (D-FM, D-C1, D-C2) and concurrent anteroposterior diameters of the medulla oblongata and upper cervical spinal cord at the corresponding anatomical levels.

**Results:**

No significant differences were observed between the PD and HC groups in OC2A or atlas posterior arch CSA (*P* > 0.05), indicating that subsequent muscle comparisons were not biased by head position or individual anatomical variation. Compared with the HC group, the PD group exhibited significantly larger CSAs of all MDBC constituent muscles (*P* < 0.05), with the most pronounced difference noted for RCPmi (PD: 1.97 ± 1.35 vs. HC: 1.58 ± 1.07, *P* = 0.003). No significant differences were found for non-constituent muscles (*P* > 0.05). Within the PD group, RCPmi CSA showed a moderate positive correlation with disease duration (*r* = 0.47, *P* < 0.001). Analysis of OAC parameters revealed significantly larger D-C1 and D-C2 distances in the PD group compared with the HC group (D-C1: 3.96 ± 1.29 mm vs. 3.60 ± 1.29 mm, *P* = 0.020; D-C2: 2.75 ± 0.88 mm vs. 2.45 ± 0.82 mm, *P* = 0.004), whereas D-FM did not differ significantly (*P* > 0.05). Concurrent measurements of neural tissue anteroposterior diameters at the same anatomical levels as the OAC parameters showed no significant differences between PD group and HC group (*P* > 0.05).

**Conclusion:**

This study demonstrates selective hypertrophy of the RCPmi in patients with PD, with the degree of hypertrophy significantly correlated with both disease duration and OAC enlargement. These morphological findings provide a cross‑sectional imaging correlate of disease chronicity. The study did not directly measure CSF dynamics or longitudinal disease progression; therefore, any link between RCPmi hypertrophy and CSF function remains hypothetical and requires future functional and longitudinal validation.

## Introduction

MDB is an anatomical structure composed of dense connective tissue that connects the spinal dura mater to the muscles and ligaments of the suboccipital region [1–6]. In 2020, Zheng et al. [7] first proposed the concept of the MDBC, defining it as a functional unit comprising the suboccipital muscles—including the RCPmi, RCPma, and OCI—together with the nuchal ligament. Fibers from this complex connect to the spinal dura mater primarily through the atlanto-axial interspace (originating from RCPmi, RCPma, and OCI) and the atlanto-occipital interspace (originating from RCPmi and RCPma).

In 2013, Sui et al. [8] first systematically described the anatomical connections between suboccipital structures and the spinal dura mater and concurrently proposed the kinetic hypothesis that contraction of the suboccipital muscles may drive CSF circulation. In 2025, Chen et al. [9] further demonstrated that the MDB is a highly conserved anatomical structure universally present in vertebrates, providing comparative anatomical evidence supporting this hypothesis. CSF circulation is regulated by multiple factors, including respiratory activity [10], arterial pulsation [11, 12], and CSF production rate [13–16]. Studies have shown that tension generated by the MDBC on the spinal dura mater during head movement acts as a biomechanical pump, significantly promoting CSF flow. Direct evidence confirming the involvement of the MDBC in regulating CSF dynamics has been established [8, 16–18].

The classic pathological feature of PD is the abnormal deposition of α-synuclein within the central nervous system, a process closely associated with neurodegeneration [19–22]. The GS serves as a crucial clearance pathway in the brain, effectively removing such pathological proteins through CSF circulation and playing a key role in maintaining brain homeostasis [23]. Multiple studies have consistently demonstrated attenuated CSF dynamics in PD patients, characterized by reduced CSF flow velocity and volume [24–26]. This dysfunction may decrease the clearance efficiency of neurotoxic proteins, thereby accelerating neurodegenerative processes. Furthermore, reduced coupling strength between CSF dynamics and global brain activity has been shown to correlate significantly with cognitive impairment in PD patients [27], suggesting that CSF dynamic parameters may serve as potential biomarkers for monitoring disease progression [28].

Concurrently, early-stage PD is frequently associated with cervical biomechanical abnormalities, such as postural instability and axial dystonia [29], the mechanisms of which may involve alterations in peripheral biomechanical structures. The MDBC is located at the craniocervical junction, with the RCPmi as its core component, directly connected to the dura mater via the myodural bridge [8, 16–18, 30, 31]. This anatomical connection plays a critical role in regulating CSF flow and mechanical force transmission at the craniocervical junction. MDBC dysfunction has been implicated in conditions such as Chiari malformation and syringomyelia [32, 33]; MRI studies have also linked RCPmi hypertrophy to chronic headache [34]. Based on this evidence, we hypothesize that the RCPmi, as the core structure of the MDBC, may participate in the pathological processes of PD through its influence on CSF dynamics.

However, elucidating the role of the MDBC in PD faces two major challenges: (1) the lack of precise methods for quantifying morphological features of MDBC-associated muscles, particularly the RCPmi, in PD patients; and (2) insufficient validation of the specific mechanisms by which the MDBC influences CSF circulation in PD models. To overcome the influence of individual anatomical variation on muscle measurements, this study introduces a standardized CSA ratio—defined as the ratio of muscle CSA to the CSA of the atlas posterior arch [35]—as an objective metric for evaluating muscle size, applied here for the first time in a PD cohort to quantitatively assess morphological changes in MDBC-associated muscles. Additionally, we focus on the OAC [36], a key region where the MDBC exerts its biomechanical effects, to investigate whether morphological alterations of the RCPmi may be associated with CSF dynamics within the OAC and thereby could theoretically contribute to the pathogenesis of PD.

This study aims to systematically elucidate the coupling mechanism between the RCPmi and CSF dynamics within the “MDBC–CSF–GS” axis, providing new insights into the roles of CSF hydrodynamics and the glymphatic system in the etiology of PD.

## Materials and methods

### Study design and ethics statement

This retrospective observational study was approved by the Institutional Review Board of Dalian Medical University (Approval No. 2024005), and all procedures were conducted in accordance with the principles of the Declaration of Helsinki.

### Data source and study population

We retrospectively enrolled 349 participants who underwent head and neck MRI examinations at the 967th Hospital between January 2016 and September 2024. The study cohort comprised:

Parkinson’s disease (PD) group: 156 patients (70 males, 86 females; age range: 45–87 years, mean age: 66.13 ± 8.36 years).

Healthy control (HC) group: 193 subjects (86 males, 107 females; age range: 59–100 years, mean age: 71.82 ± 8.30 years).

According to the World Health Organization age classification criteria [37], participants were divided into two subgroups: ≤65 years (younger group) and > 65 years (older group).

### Inclusion and exclusion criteria

Inclusion criteria for the PD group: (1) For the PD group, diagnoses fulfilling the Chinese Diagnostic Criteria for Parkinson’s Disease (2016 Edition) [38]; (2) Age ≥ 45 years; (3) Complete head and neck MRI data available.

Inclusion criteria for the HC group: (1) Age and sex matched to the PD group; (2) MRI or CT findings showing only age-related cerebral changes, with no evidence of cerebral infarction, intracranial space-occupying lesions, or other neurological diseases; (3) No documented history of central nervous system disease.

Exclusion criteria (applicable to both groups): (1) History of stroke, traumatic brain injury, central nervous system infection, other intracranial structural lesions, psychiatric disorders, hydrocephalus, or severe cardiovascular disease; (2) History of cervical spine surgery; (3) Poor quality or incomplete MRI scans (e.g., motion artifacts, missing key slices). Additionally, all healthy controls were rigorously screened to exclude clinically confirmed cervical spondylosis or chronic neck pain, thereby eliminating the potential influence of these common conditions on suboccipital muscle morphology.

### Image acquisition

All MRI scans were performed using a Siemens Spectra 3.0T superconducting magnetic resonance scanner (Siemens Healthineers, Germany) with a head-neck coil. Participants were placed in a standard supine position with the head positioned in the coil, and a laser localizer was used to ensure the Frankfurt plane was perpendicular to the examination table, minimizing the effect of neck flexion/extension on muscle measurements. Axial T2-weighted imaging of the head was performed using a fast spin-echo sequence with the following parameters: repetition time 3500 ms, echo time 90 ms, field of view 24 cm × 24 cm, matrix 256 × 256, slice thickness 5 mm, interslice gap 1 mm, and number of signal acquisitions 2.

### Image measurement methods

All imaging measurements were performed on a picture archiving and communication system (PACS) workstation. Two neuroradiologists, blinded to participant group allocation, independently performed all measurements. Each parameter was measured twice, and the mean values were used for final statistical analysis.

#### Measurement of occiput-C2 angle (*n* = 349)

**Fig. 1:**
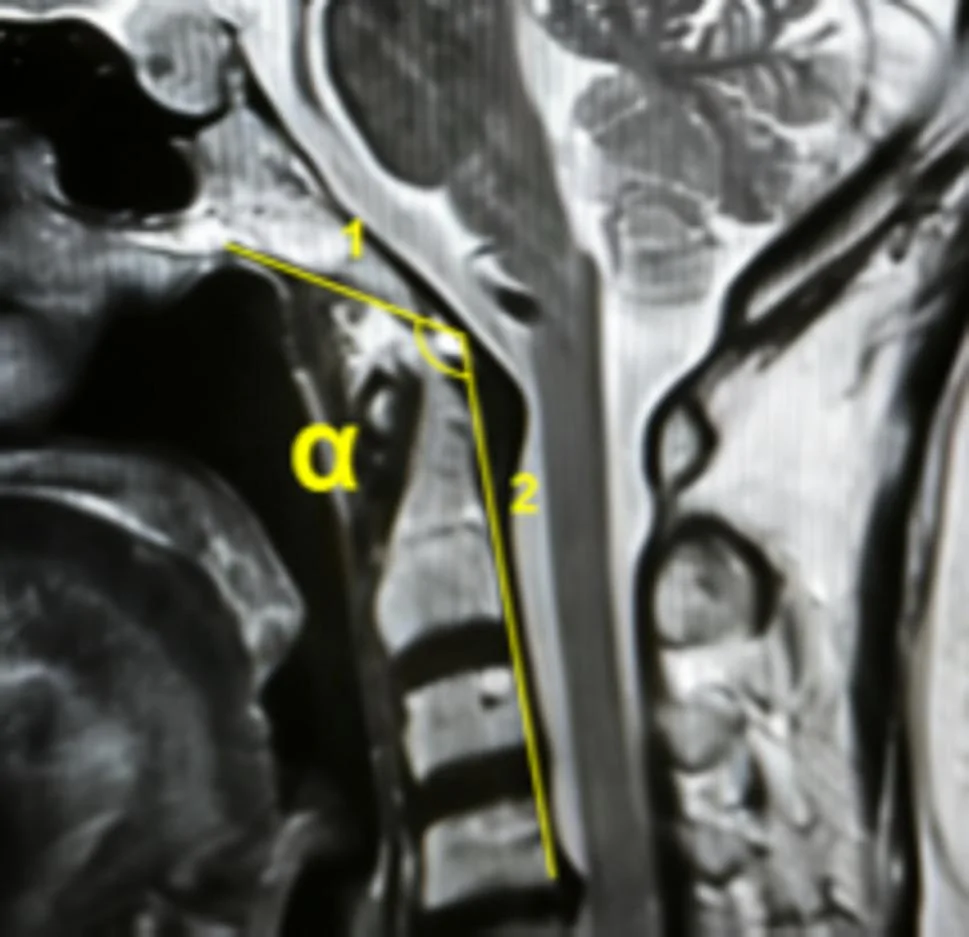
Measurement of OC2A on a mid‑sagittal T2‑weighted image of the head and neck. Reference line 1 is drawn along the posterior margin of the clivus. Reference line 2 is drawn as a tangent along the posterior margin of the second cervical vertebra (C2). The α angle represents the (OC2A), defined as the angle between these two reference lines

To eliminate potential confounding effects of head position on muscle morphometry, the occiput-C2 angle (OC2A) was measured on mid-sagittal T2-weighted images. The measurement method was as follows (Fig. 1).

#### Morphometric analysis of MDBC muscles (*n* = 349)

**Fig. 2:**
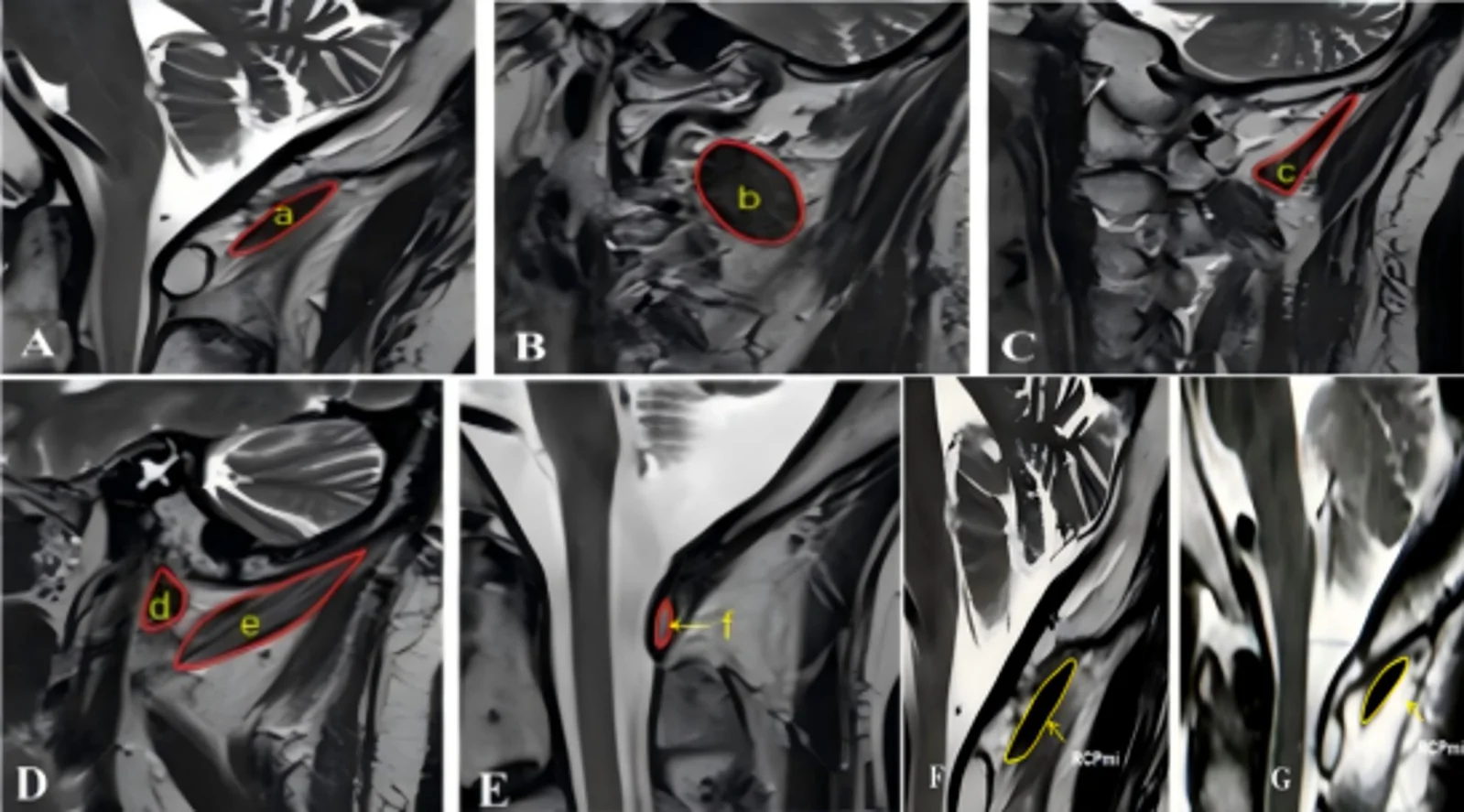
Measurement of the CSA of MDBC constituent and non‑constituent muscles, and the atlas posterior arch used for normalization. Sagittal T2‑weighted images show the maximum CSA delineated by red outlines for: (**a**) rectus capitis posterior minor (RCPmi); (**b**) obliquus capitis inferior (OCI); (**c**) rectus capitis posterior major (RCPma); (**d**) rectus capitis lateralis (RCL); (**e**) obliquus capitis superior (OCS); and (**f**) the posterior arch of the atlas. The normalized CSA for each muscle was calculated as the ratio of the individual muscle CSA (**a**–**e**) to the CSA of the atlas posterior arch (**f**) to correct for individual anatomical variation. Representative examples of maximum CSA measurements for the RCPmi are shown for a HC (**F**) and a patient with PD (**G**)

**Internal reference validation** The cross-sectional area (CSA) of the cancellous the atlas posterior arch(excluding cortical bone and intramuscular fat) was measured on mid-sagittal T2-weighted images in all participants. Values were compared between the PD and HC groups to verify the stability of this structure as an internal reference (Fig. 2-E). Although bone area can be influenced by age, sex, and disease, the atlas posterior arch cancellous bone was selected as the normalization reference because: (i) it is directly adjacent to the target muscles (RCPmi, RCPma, OCI) and shares the same local biomechanical environment; (ii) it is not a primary site of degenerative change in PD; and (iii) our empirical data confirmed no significant differences in its CSA between PD and HC groups in overall, age‑stratified, or sex‑stratified comparisons (Table 3). Thus, it serves as a stable internal reference independent of disease status [35].

**Standardization based on validation** Based on the above validation, the standardized CSA ratio was adopted as the primary analytical metric, calculated as: CSA = Net CSA of target muscle / Cancellous bone CSA of the atlas posterior arch in the same individual [35]. This ratio effectively corrects for confounding biases arising from individual variations in body size, thereby enabling a more precise assessment of the specific association between PD and morphological alterations in specific muscles, particularly the rectus capitis posterior minor (RCPmi).

**Muscle measurement method** On consecutive axial T2-weighted images, the slice with the clearest muscle boundaries was selected for measurement, and all measurements were performed on planes perpendicular to the long axis of each muscle. To minimize the influence of intramuscular fat infiltration, the CSA was measured: within manually delineated regions of interest, high‑signal areas corresponding to fatty tissue were excluded using signal intensity threshold segmentation. The maximum CSA of each muscle was measured bilaterally, and the average of the bilateral values was used for final analysis. The muscles measured included the MDBC constituent muscles—RCPmi, RCPma and OCI—and the control (non‑constituent) muscles—OCS and RCL (Fig. 2‑A–E).

Representative examples of maximum CSA measurements for the RCPmi in a HC and a patient with PD are shown in Fig. 2‑F and G, respectively.

### Morphometric analysis of the occipito-atlantal cistern(OAC)(*n* = 277)

Definition of Regular OAC Morphology On mid-sagittal T2-weighted images, regular OAC morphology was defined by continuous CSF signal without local adhesions, septations, or mass effects. Based on this criterion, 277 participants were selected from the initial 349 for subgroup analysis, including 114 PD patients (53 males, 61 females; mean age 66.59 ± 8.55 years) and 163 healthy controls (74 males, 89 females; mean age 71.63 ± 8.09 years).

Following the method described by Li et al. [36], OAC volumetric parameters were measured on mid‑sagittal T2‑weighted images as follows:

D‑FM: the anteroposterior dimension of the dorsal subarachnoid space at the level of the foramen magnum, measured from the posterior margin of the foramen magnum to the dorsal surface of the medulla oblongata.

D‑C1: the anteroposterior dimension of the dorsal subarachnoid space at the level of the atlas (C1), measured from the inner convexity of the posterior arch of C1 to the dorsal surface of the spinal cord.

D‑C2: the anteroposterior dimension of the dorsal subarachnoid space at the level of the axis (C2), measured from the inner convexity of the lamina of C2 to the dorsal surface of the spinal cord.

**Fig. 3:**
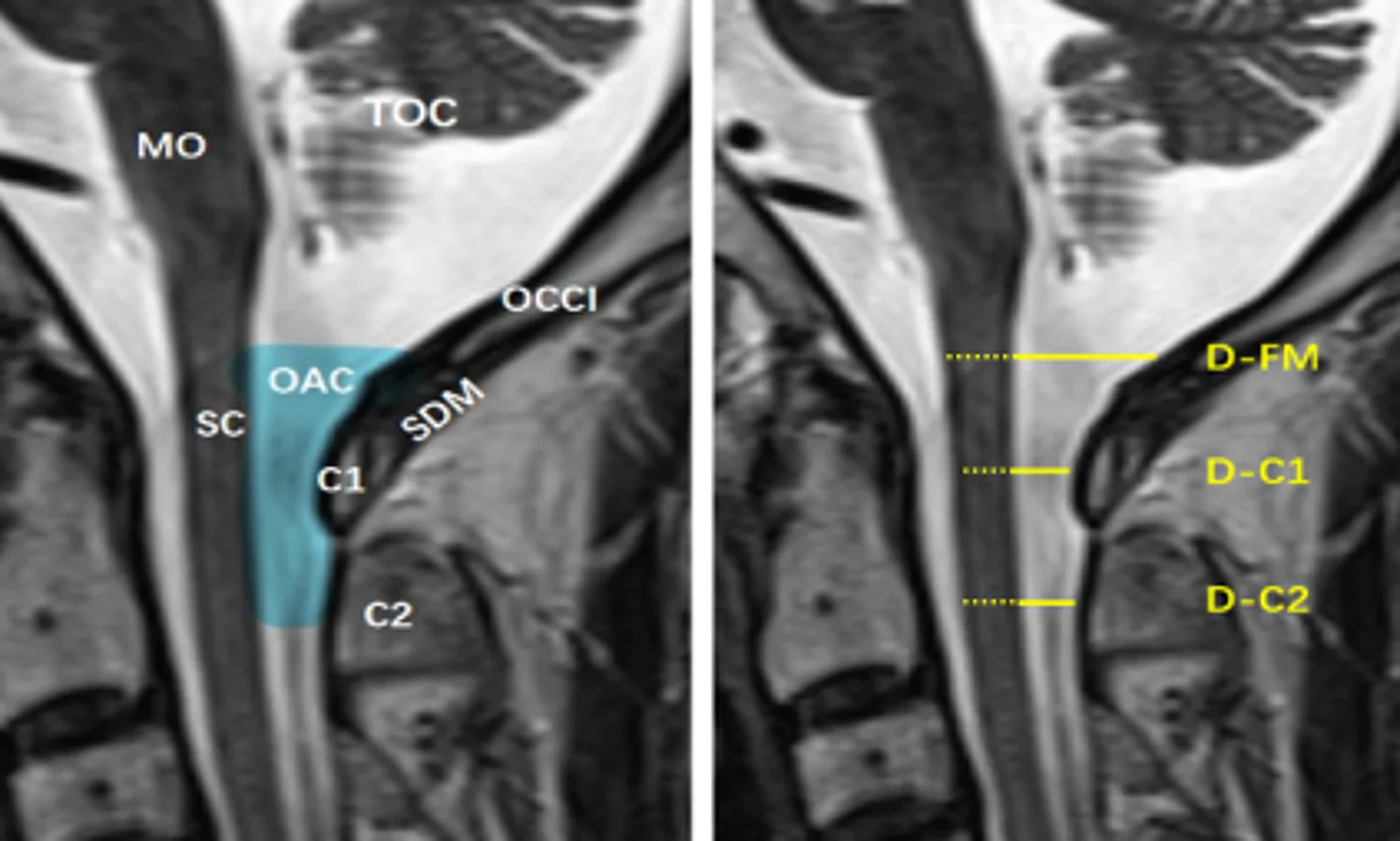
Measurement of OAC volumetric parameters and corresponding anteroposterior diameters of the medulla oblongata and upper cervical spinal cord on a mid‑sagittal T2‑weighted image. Left panel: The OAC anatomy is indicated by the blue overlay, defined as the dorsal subarachnoid space between the medulla oblongata/spinal cord and the posterior elements of the foramen magnum, atlas (C1), and axis (C2). Right panel: The yellow overlays illustrate the method for measuring OAC volumetric parameters. OAC dimensions were assessed using three linear distances: D‑FM、D‑C1 and D‑C2. Measurement of neural tissue anteroposterior diameters: At the same anatomical levels as the OAC parameters (D‑FM, D‑C1, D‑C2; indicated by the yellow dashed lines), the anteroposterior diameters of the medulla oblongata and upper cervical spinal cord were concurrently measured to evaluate the potential contribution of neural tissue atrophy to OAC enlargement

To assess the potential contribution of neural tissue atrophy to OAC enlargement, the anteroposterior diameters of the medulla oblongata and upper cervical spinal cord (at the C1–C2 level) were concurrently measured on mid‑sagittal T2‑weighted images at the same anatomical levels as the OAC parameters (D‑FM, D‑C1, D‑C2) **(**Fig. 3**)**.

All measurements were independently performed by two radiologists blinded to group allocation, and the mean values were used for final analysis. Inter‑ and intra‑observer reliability were assessed using the intraclass correlation coefficient (ICC).

### Clinical data collection

Information on disease onset was obtained through telephone follow-up of all 156 PD patients. Due to the inherent limitations of the retrospective design, precise onset dates were not clearly documented in the medical records of 105 patients. Consequently, 51 patients with reliable disease duration data were included in this subgroup analysis (27 males, 24 females; disease duration range: 1–14 years, mean: 3.75 ± 3.18 years). To assess the representativeness of this subgroup, age, sex distribution, and RCPmi standardized net area were compared between the included and excluded patients; no statistically significant differences were found (*P* > 0.05).

### Inter- and intra-observer reliability assessment

Forty participants were randomly selected for reliability analysis. Two independent raters performed all measurements, and one rater repeated the measurements after a two-week interval. Intraclass correlation coefficients (ICCs) were calculated to assess inter- and intra-observer reliability. ICC > 0.75 was considered good reliability, and ICC 0.60–0.75 was considered moderate reliability.

### Statistical analysis

Statistical analysis was performed using SPSS software (version 27.0, IBM Corp., Armonk, NY, USA). Continuous variables are presented as mean ± standard deviation, and categorical variables are presented as frequencies (percentages). Normality was assessed using the Shapiro-Wilk test.

Group comparisons: Independent samples t-tests were used for normally distributed continuous variables; Mann-Whitney U tests were used for non-normally distributed variables; chi-square tests were used for categorical variables; one-way ANOVA with Bonferroni correction for post-hoc analysis was used for multiple group comparisons.

Correlation analysis: Pearson correlation was used for normally distributed data, and Spearman rank correlation was used for non-normally distributed data. Multiple linear regression models were employed to adjust for potential confounding factors, including age, sex, and OC2A, on the primary outcome measures.

All tests were two-tailed, and(*P* < 0.05) was considered statistically significant.

## Results

### Sample characteristics

#### Demographic characteristics

The final analysis included 349 participants (PD: *n* = 156; HC: *n* = 193), stratified by age (≤ 65 years: younger subgroup; >65 years: older subgroup) and sex. Chi-square tests revealed no significant differences in age (*P* = 0.512) or sex distribution (*P* = 0.953) between the PD and HC groups. From this cohort, 277 participants with morphologically regular occipito-atlantal cisterns (OAC) (PD: *n* = 114; HC: *n* = 163) were selected for OAC morphological analysis, similarly stratified by age and sex. Chi-square tests indicated no significant differences in age (*P* = 0.593) or sex distribution (*P* = 0.857) between the PD and HC groups within this OAC subgroup (Table 1.1).

**Table 1.1:** Descriptive analysis of patients with Parkinson’s disease (PD) and healthy controls (HC)

Group	Variable	Category	Total Sample (*n* = 349)		P Value	OAC Sample(*n* = 277)		P Value
			PD(*n* = 156)	HC (*n* = 193)		PD (*n* = 114)	HC (*n* = 163)	
PD	Sex	Male	70 (44.9%)			53 (46.5%)		
		Female	86 (55.1%)			61 (53.5%)		
PD	Age	≤ 65 y	52 (33.3%)			37 (32.5%)		
		> 65 y	104 (66.7%)			77 (67.5%)		
HC	Sex	Male		86 (44.6%)			74 (45.4%)	
		Female		107 (55.4%)			89 (54.6%)	
HC	Age	≤ 65 y		58 (30.1%)			48 (29.4%)	
		> 65 y		135 (69.9%)			115(70.6%)	
Total	Sex	Male	156 (44.7%)		0.953	127 (45.8%)		0.857
		Female	193 (55.3%)			150 (54.2%)		
Total	Age	≤ 65 y	110 (31.5%)		0.512	85 (30.7%)		0.593
		> 65 y	239 (68.5%)			192 (69.3%)		

Note: Data are presented as n (%). Chi-square tests demonstrated no statistically significant differences in sex or age distribution between PD patients and healthy controls in either the total sample or the OAC subsample (*P* > 0.05). Percentages are calculated within each group

#### Inter-rater reliability assessment

The intraclass correlation coefficients (ICCs) for all measurements showed excellent inter‑rater reliability. Specific ICC values are reported (Table 1.2).

**Table 1.2:** Intraclass correlation coefficients for each measurement parameter

Measurement parameter	ICC	95% CI	*P* value
Occiput-C2 angle	0.92	0.85–0.96	< 0.001
Atlas posterior arch CSA	0.89	0.80–0.94	< 0.001
RCPmi CSA	0.82	0.65–0.93	< 0.001
RCPma CSA	0.94	0.89–0.97	< 0.001
OCI CSA	0.91	0.77–0.97	< 0.001
OCS CSA	0.88	0.79–0.93	< 0.001
RCL CSA	0.93	0.87–0.96	< 0.001
D-FM	0.84	0.72–0.94	< 0.001
D-C1	0.90	0.83–0.94	< 0.001
D-C2	0.88	0.65–0.96	< 0.001
Neura Tissue Anteroposterior(AP) Diameters			
Same level as D-FM	0.85	0.71–0.93	< 0.001
Same level as D-C1	0.87	0.76–0.94	< 0.001
Same level as D-C2	0.89	0.81–0.95	< 0.001

Note: ICCs were calculated from a random sample of 40 participants

### Analysis of the occiput-C2 angle (OC2A)

To account for potential confounding effects of head positioning on muscle morphometry, the OC2A was measured on mid-sagittal T2-weighted images in all 349 participants. No statistically significant difference in OC2A was observed between the PD and HC groups (PD: 128.72 ± 9.41° vs. HC: 130.60 ± 8.18°, *P* = 0.313), indicating that subsequent comparisons of muscle morphology were not biased by variations in head position (Table 2).

**Table 2:** Comparison of the occiput-C2 angle (OC2A) between the Parkinson’s disease (PD) and healthy control (HC) groups (*N* = 349)

Parameter	PD Group (*n* = 156)	HC Group(*n* = 193)	t value	*P* Value
Occiput-C2 angle (°)	128.72 ± 9.41	130.60 ± 8.18	1.014	0.313

Note: Data are presented as mean ± standard deviation. A P value < 0.05 was considered statistically significant. Abbreviations: PD, Parkinson’s disease; HC, healthy control

### Internal reference validation

No statistically significant difference was observed in the cancellous bone area of the atlas posterior arch between the PD and HC groups (*P* > 0.05), confirming its structural stability and suitability as a normalization factor independent of disease status. Based on this validation, the standardized cross-sectional area (CSA) ratio for each target muscle was calculated. This normalization effectively corrects for inter-individual anatomical variation (e.g., body size, skeletal dimensions), thereby enabling a more accurate assessment of the specific association between PD and morphological alterations in MDBC constituent muscles, particularly the RCPmi (Table 3).

**Table 3:** Demographic characteristics and comparison of atlas posterior arch cross-sectional area between Parkinson’s disease patients and healthy controls (*N* = 349)

Variable/Subgroup	PD Group (*n* = 156)	HC Group(*n* = 193)	t value	*P* Value
**Overall comparison**				
Area (mm²)	31.33 ± 17.17	31.73 ± 19.58	–0.20	0.841
**Stratified by age**				
≤ 65 years (mm²)	30.87 ± 16.54 (*n* = 55)	32.15 ± 19.82 (*n* = 65)	–0.39	0.696
> 65 years (mm²)	31.58 ± 17.56 (*n* = 101)	31.52 ± 19.48 (*n* = 128)	0.03	0.980
**Stratified by sex**				
Male (mm²)	32.54 ± 18.23 (*n* = 76)	32.68 ± 20.71 (*n* = 92)	–0.05	0.963
Female (mm²)	30.18 ± 16.14 (*n* = 80)	30.86 ± 18.51 (*n* = 101)	–0.26	0.794

Note: Data are presented as mean ± SD, with subgroup sample sizes indicated in parentheses (stratified analyses only). A P value < 0.05 was considered statistically significant. Abbreviations: PD, Parkinson’s disease; HC, healthy control

### Comparison of MDBC muscle cross-sectional areas

#### Overall comparison between PD and HC groups

The PD group exhibited significantly larger standardized net areas of all MDBC constituent muscles—RCPmi, RCPma, and OCI—compared to the HC group (all *P* < 0.05). This difference was most pronounced for RCPmi (PD: 1.97 ± 1.35 vs. HC: 1.58 ± 1.07, *P* = 0.003). In contrast, no significant differences were observed between groups for the non-constituent muscles OCS (PD: 7.15 ± 4.44 vs. HC: 7.50 ± 5.41, *P* = 0.513) and RCL (PD: 3.34 ± 2.26 vs. HC: 3.64 ± 2.40, *P* = 0.226) (Table 4.1).

**Table 4.1:** Comparison of cross-sectional area (CSA) of MDBC constituent and non-constituent muscles in total sample (*N* = 349)

Muscle	PD(*n* = 156)	HC(*n* = 193)	t value	*P* value
**Constituent muscles**				
RCPmi	1.97 ± 1.35	1.58 ± 1.07	3.04	**0.003†**
RCPma	6.91 ± 4.00	6.37 ± 4.19	1.22	0.223
OCI	9.18 ± 5.42	8.80 ± 5.62	0.64	0.526
**Non-constituent muscles**				
OCS	7.15 ± 4.44	7.50 ± 5.41	–0.66	0.513
RCL	3.34 ± 2.26	3.64 ± 2.40	–1.21	0.226

Note: Data are presented as mean ± SD. Bold P values indicate *P* < 0.05 (uncorrected). **†** After Bonferroni correction for five comparisons (RCPmi, RCPma, OCI, OCS, RCL; adjusted threshold *P* < 0.01), only RCPmi remained significant (*P* = 0.003)

#### Subgroup analysis by sex and age

Within the PD group, men exhibited significantly larger CSA for all MDBC constituent muscles compared to women (RCPmi: men 2.34 ± 1.67 vs. women 1.67 ± 0.93, *P* = 0.003; RCPma: men 7.65 ± 4.39 vs. women 6.30 ± 3.56, *P* = 0.036; OCI: men 10.51 ± 6.31 vs. women 8.09 ± 4.31, *P* = 0.005). No significant differences were observed between younger (≤ 65 years) and older (> 65 years) age subgroups within either the PD or HC group for any of the MDBC constituent muscles (all *P* > 0.05) (Table 4.2).

**Table 4.2:** Comparison of MDBC constituent muscle cross-sectional area (CSA) by sex and age subgroups

Group	Subgroup	*n*	RCPmi	*P*	RCPma	*P*	OCI	*P*
HC(*n* = 193)	Male	86	1.61 ± 1.02	0.675	6.19 ± 3.72	0.603	9.84 ± 6.39	**0.025**
HC(*n* = 193)	Female	107	1.55 ± 1.12		6.51 ± 4.55		7.96 ± 4.79	
HC(*n* = 193)	≤ 65y	58	1.66 ± 1.13	0.457	7.12 ± 4.32	0.101	9.58 ± 6.14	0.209
HC(*n* = 193)	> 65y	135	1.54 ± 1.05		6.04 ± 4.11		8.47 ± 5.38	
PD(*n* = 156)	Male	70	2.34 ± 1.67	**0.003**	7.65 ± 4.39	**0.036**	10.51 ± 6.31	**0.005**
PD(*n* = 156)	Female	86	1.67 ± 0.93		6.30 ± 3.56		8.09 ± 4.31	
PD(*n* = 156)	≤ 65y	52	1.84 ± 1.01	0.337	6.80 ± 2.79	0.819	9.30 ± 4.16	0.848
PD(*n* = 156)	> 65y	104	2.04 ± 1.50		6.96 ± 4.49		9.12 ± 5.97	

Note: Data are presented as mean ± SD. *P* < 0.05 indicates significant difference between subgroups (bold)

### Correlation between PD disease duration and RCPmi hypertrophy

#### Representativeness analysis of the disease duration subgroup

To assess potential selection bias, we compared the 51 PD patients with complete disease duration data against the 105 PD patients without such data. No statistically significant differences were observed between the two subgroups in terms of age, sex distribution, or RCPmi standardized net area (all *P* > 0.05), confirming that the 51‑patient subgroup was representative of the overall PD cohort (Table 5.1).

**Table 5.1:** Representativeness analysis of the disease duration subgroup

Variable	PD with Documented Disease Duration (*n* = 51)	PD without Documented Disease Duration (*n* = 105)	t Value	*P* Value	95% CI
Sex, n (%)					
Male, n(%)	23(45.10%)	47(44.76%)	0.018	0.894	-16.23%~17.09%
Female, n(%)	28(54.90%)	58(55.24%)			
Age group, n (%)					
≤ 65 years, n(%)	22(43.14%)	45(42.86%)	0.023	0.879	−17.31%~17.87%
>65 years, n(%)	29(56.86%)	60(57.14%)			
RCPmi CSA (mean ± SD)	1.82 ± 0.97	1.76 ± 0.89	0.352	0.725	−0.24%~0.36%

Note: Data are presented as n (%) or mean ± SD. *P* < 0.05 was considered statistically significant

#### Correlation between disease duration and RCPmi hypertrophy

A moderate positive correlation was identified between disease duration and RCPmi net area in the 51 PD patients (*r* = 0.47, *P* < 0.001), indicating that longer disease duration was associated with more pronounced RCPmi hypertrophy (Table 5.2).

**Table 5.2:** Correlation between disease duration and RCPmi cross-sectional area (CSA) in PD patients (*n* = 51)

Variable	*r* value	*P* value
Disease duration	0.47	**< 0.001**

Note: Pearson correlation coefficient. *P* < 0.05 indicates statistical significance (bold). PD, Parkinson’s disease; RCPmi, rectus capitis posterior minor

### Morphometric analysis of the occipito-atlantal cistern and neural tissue

#### Comparison of OAC parameters and corresponding neural tissue anteroposterior diameters between PD and HC groups

Among the 277 participants with well-defined OAC morphology, the PD group exhibited significantly larger D-C1 and D-C2 distances compared to the HC group (D-C1: 3.96 ± 1.29 mm vs. 3.60 ± 1.29 mm, *P* = 0.020; D-C2: 2.75 ± 0.88 mm vs. 2.45 ± 0.82 mm, *P* = 0.004). No significant difference was observed in D-FM between the two groups (*P* > 0.05).

Concurrent measurements of neural tissue anteroposterior diameters at the same anatomical levels as the OAC parameters revealed no significant differences between PD patients and HC subjects (*P* > 0.05). These findings suggest that enlargement of the OAC in PD patients tends to be driven primarily by mechanical traction, with only a limited contribution from neural tissue atrophy (Table 6.1).

**Table 6.1:** Comparison of occipito-atlantal cistern (OAC) parameters and corresponding neural tissue anteroposterior diameters between PD and HC groups

Parameter(mm)	PD Group(*n* = 114)	HC Group(*n* = 163)	t value	*P* value
**OAC parameters**				
D-FM	13.34 ± 3.17	13.28 ± 2.58	0.16	0.876
D-C1	3.96 ± 1.29	3.60 ± 1.29	2.33	**0.020**
D-C2	2.75 ± 0.88	2.45 ± 0.82	2.89	**0.004‡**
**Neural Tissue Anteroposterior(AP) Diameters**				
Same level as D-FM	8.92 ± 2.61	9.21 ± 1.93	-0.602	0.549
Same level as D-C1	6.06 ± 1.21	6.64 ± 2.03	-1.620	0.110
Same level as D-C2	5.40 ± 1.03	5.37 ± 1.45	0.114	0.910

Note: Data are presented as mean ± SD (mm). Bold P values indicate *P* < 0.05 (uncorrected). AP, anteroposterior. **‡** After Bonferroni correction for three OAC parameters (adjusted threshold *P* = 0.05/3 ≈ 0.0167), D‑C2 remained significant (*P* = 0.004); D‑C1 (*P* = 0.020) did not survive correction. Neural tissue comparisons are exploratory and were not corrected for multiple comparisons

#### Subgroup analysis of OAC parameters by sex and age

Subgroup analyses revealed significant sex-based differences in D-FM within both the PD and HC groups, with males exhibiting larger values than females (PD: 14.34 ± 3.32 mm vs. 12.56 ± 2.88 mm, *P* = 0.003; HC: 14.06 ± 2.83 mm vs. 12.63 ± 2.16 mm, *P* < 0.001). No significant sex differences were observed for D-C1 or D-C2 in either group.

Age-related comparisons demonstrated that older PD patients (> 65 years) had significantly larger D-C1 and D-C2 distances compared to younger PD patients (≤ 65 years) (D-C1: 4.03 ± 1.29 mm vs. 3.79 ± 1.27 mm, *P* = 0.005; D-C2: 2.88 ± 0.91 mm vs. 2.43 ± 0.76 mm, *P* = 0.010). In the HC group, a significant age-related difference was observed only for D-C1 (older: 3.59 ± 1.25 mm vs. younger: 3.62 ± 1.18 mm, *P* = 0.018) (Table 6.2).

**Table 6.2:** Subgroup analysis of OAC parameters by sex and age

Group	Subgroup	*n*	D-FM(mm)	*P* value	D-C1(mm)	*P* value	D-C2(mm)	*P* value
PD(*n* = 114)	Male	53	14.34 ± 3.32	**0.003**	3.85 ± 1.26	0.432	2.82 ± 0.91	0.333
PD(*n* = 114)	Female	61	12.56 ± 2.88		4.04 ± 1.30		2.66 ± 0.86	
PD(*n* = 114)	≤ 65y	37	12.90 ± 2.67	0.265	3.79 ± 1.27	**0.005**	2.43 ± 0.76	**0.010**
PD(*n* = 114)	> 65y	77	13.62 ± 3.42		4.03 ± 1.29		2.88 ± 0.91	
HC(*n* = 163)	Male	74	14.06 ± 2.83	**< 0.001**	3.76 ± 1.31	0.140	2.58 ± 0.97	0.078
HC(*n* = 163)	Female	89	12.63 ± 2.16		3.47 ± 1.14		2.34 ± 0.67	
HC(*n* = 163)	≤ 65y	48	13.21 ± 2.38	0.832	3.62 ± 1.18	**0.018**	2.35 ± 0.79	0.313
HC(*n* = 163)	> 65y	115	13.31 ± 2.67		3.59 ± 1.25		2.49 ± 0.84	

Note: Data are presented as mean ± SD (mm). *P* < 0.05 indicates significant difference between subgroups (bold)

## Discussion

### Summary of main findings

This study conducted a retrospective analysis of head and neck MRI data from 349 participants, comprising 156 patients with PD and 193 HC. The main findings are as follows: (1) No significant differences in OC2A or atlas posterior arch CSA were observed between PD and HC groups, confirming that subsequent muscle comparisons were not biased by head position or individual anatomical variation. (2) PD patients exhibited selective hypertrophy of MDBC constituent muscles, with RCPmi hypertrophy being most pronounced and showing a moderate positive correlation with disease duration. (3) PD patients demonstrated significant enlargement of the OAC, reflected by increased D-C1 and D-C2 distances. (4) Concurrent measurements of neural tissue anteroposterior diameters at the same anatomical levels as the OAC parameters revealed no significant differences between PD and HC groups. (5) Sex- and age-related differences were observed in both muscle CSA and OAC parameters. These findings provide new imaging evidence for understanding peripheral biomechanical alterations in PD. It is important to emphasize that all findings are anatomical and imaging‑based; the proposed links to CSF dynamics or glymphatic function remain hypothetical and require direct functional validation (e.g., phase‑contrast MRI or DTI‑ALPS).

### RCPmi hypertrophy: specific association and potential clinical significance

The core pathological feature of PD is the abnormal deposition of pathological proteins such as α-synuclein in the brain, a process closely linked to impaired clearance function of the glymphatic system for metabolic waste products [19–22]. Abnormalities in CSF dynamics have been implicated in the development and progression of non-motor symptoms in PD, such as cognitive impairment [27]. Notably, early-stage PD patients often present with motor symptoms including reduced cervical range of motion, postural instability, and axial dystonia [29]; these clinical manifestations cannot be fully explained by central pathology alone, suggesting the involvement of peripheral biomechanical structures. As a core component of the MDBC, the RCPmi, due to its unique anatomical features—with some fibers traversing the atlanto-occipital interspace to fuse with the spinal dura mater [30, 31, 39, 40]—is hypothesized to participate in the regulation of CSF flow [8, 16–18]. Histologically, the RCPmi exhibits the largest CSA of slow-twitch fibers and the highest density of muscle spindles per unit mass among the suboccipital muscles [41, 42]. These anatomical and histological characteristics underpin the central role of the RCPmi within the biomechanical system of the MDBC.

This study quantitatively assessed the morphological characteristics of MDBC constituent and non-constituent control muscles. The results showed no significant differences in the net CSA of non-MDBC muscles (OCS, RCL) between the PD and HC groups (OCS: *P* = 0.513; RCL: *P* = 0.226), indicating that their morphology is unrelated to PD. In contrast, the PD group exhibited increased net CSA in all MDBC constituent muscles, with the most pronounced increase observed in the RCPmi (PD group: 1.97 ± 1.35 mm² vs. HC group: 1.58 ± 1.07 mm², *P* = 0.003). This finding suggests a specific association between MDBC constituent muscles, particularly the RCPmi, and PD.

Our analysis confirmed that the subgroup of 51 patients with complete disease duration records was representative of the overall PD cohort. Further analysis revealed a moderate positive correlation between PD disease duration and RCPmi net CSA (*r* = 0.47, *P* < 0.001), indicating that RCPmi hypertrophy becomes more pronounced with longer disease duration. This finding suggests that morphological changes in the RCPmi may be associated with disease duration and hold potential as a cross‑sectional imaging correlate. RCPmi size showed no significant correlation with patient age (*P* = 0.337) but exhibited a significant sex difference: male PD patients had significantly larger RCPmi net areas than female patients (*P* = 0.003), a trend consistent with findings in healthy populations [43–45]. While sex is a recognized factor influencing muscle morphology and disease manifestation, its direct link to the pathogenesis of PD requires further validation through longitudinal studies. Although the 51‑patient subgroup was representative of the overall cohort in age, sex, and RCPmi CSA, the relatively small sample size for the correlation analysis warrants cautious interpretation. The moderate correlation (*r* = 0.47) should be confirmed in larger, prospective cohorts before any firm conclusions about disease‑duration dependency can be drawn.

### OAC enlargement: traction effect versus atrophic effect

The occipito-atlantal cistern (OAC) [36] is a key CSF space adjacent to the MDBC and represents a core dynamic region where MDBC-driven CSF flow occurs [8, 37]. This study found significantly larger D-C1 and D-C2 distances in the PD group compared with the HC group, indicating abnormal OAC enlargement in PD patients.

Regarding the mechanism of OAC enlargement, two possible explanations warrant consideration:

First, the mechanical traction effect. Previous studies have suggested that OAC enlargement may be related to altered dural compliance secondary to increased mechanical loading from the MDBC, particularly a hypertrophic RCPmi [46, 47]. Speculatively, RCPmi hypertrophy could theoretically increase cyclic traction on the dura mater, but this hypothesis lacks direct functional evidence and requires testing with phase‑contrast MRI.

Second, the ex-vacuo phenomenon. PD and atypical parkinsonian syndromes are often accompanied by atrophy of the medulla oblongata and upper cervical spinal cord. To assess this possibility, this study concurrently measured neural tissue anteroposterior diameters at the same anatomical levels as the OAC parameters. The results showed no significant differences between PD and HC groups (*P* > 0.05), a finding that does not support the ex-vacuo phenomenon as the primary mechanism underlying OAC enlargement. Therefore, the cross-sectional data from this study tend to favor the mechanical traction effect as the predominant contributor to OAC enlargement.

Unlike type I Chiari malformation, which is typically characterized by OAC stenosis and increased CSF flow velocity [48, 49]. The OAC enlargement observed in our PD patients might be associated with reduced CSF flow velocity. This difference suggests that the pathophysiological significance of morphological changes in the OAC may vary across different disease states.

### Alternative explanation: muscle adaptation secondary to motor symptoms

It is important to note that the absence of quantitative assessments for cervical dystonia, postural abnormalities, or the severity of motor symptoms presents an alternative, more direct clinical explanation for the observed RCPmi hypertrophy. PD patients frequently exhibit cervical dystonia and postural abnormalities, leading to chronic isometric contraction of the neck muscles. This chronic increase in workload could itself induce compensatory muscle hypertrophy, analogous to exercise-induced muscle enlargement.

As a preliminary exploration of this possibility, the OC2A was measured and compared between groups; the lack of significant difference (*P* > 0.05) indicates that confounding by head position was effectively controlled. However, due to the limitations of the retrospective design, UPDRS-III neck rigidity scores were not systematically collected for all patients, preventing a direct assessment of the association between the degree of neck rigidity and RCPmi hypertrophy. Distinguishing whether RCPmi hypertrophy represents a primary participant in the pathological process of PD or a secondary manifestation of its motor symptoms will require prospective longitudinal studies.

### Age and sex effects

This study found that older PD patients (> 65 years) had significantly higher D-C2 values compared with younger PD patients (*P* = 0.01). This phenomenon may be related to age-related structural evolution of the MDBC [50], although this requires further validation. The OAC parameter D-FM showed significant sex differences in both the PD and HC groups (males > females; PD group: *P* = 0.003; HC group: *P* < 0.001), which contrasts with the report by Li et al. [36] stating no sex difference in OAC size. Given the larger sample size of the present study (*n* = 349 vs. *n* = 120) and the consistent observation of sex differences across both groups, we consider our findings to be more robust. The mechanism underlying these sex differences remains unclear but may be related to larger bony structures and greater muscle mass at the craniocervical junction in males.

### Mechanistic hypothesis: a working model requiring validation

The following discussion of potential mechanisms is speculative and intended to generate hypotheses for future studies; no causal claims are made. Integrating the established theory that the MDBC drives CSF circulation [8, 16–18], findings from animal studies demonstrating that pathological hypertrophy of the RCPmi can disrupt CSF secretion-reabsorption balance [51], and the morphological observations from the present study—particularly the key finding of OAC enlargement without a corresponding reduction in spinal cord diameter—we propose the following working hypothesis: Pathological hypertrophy of the RCPmi may be associated with alterations in the local CSF dynamic environment, which could theoretically interfere with the clearance efficiency of the glymphatic system for neurotoxic metabolites, although this has not been directly measured. This hypothesis encompasses two potential levels: (1) short-term mechanical effects, where increased tension generated by the hypertrophic RCPmi is transmitted via the MDBC to the spinal dura mater, altering the local CSF pressure gradient within the OAC; and (2) long-term structural remodeling, where sustained mechanical load imbalance may induce fibrotic changes in the dura mater, reducing tissue compliance. It is important to emphasize that the mechanisms described above remain speculative and lack direct functional evidence; they require validation in future studies incorporating quantitative measurements of CSF flow, such as phase-contrast MRI.

### Limitations

This study has several limitations that should be acknowledged.

First, **slice thickness limitations.** The use of a 5 mm slice thickness in this study, dictated by the routine clinical MRI protocol, is suboptimal for morphometric analysis of small structures like the RCPmi and may introduce partial volume effects, potentially biasing absolute measurement values. Moreover, the limited spatial resolution inherent to conventional 5-mm-thick acquisitions may reduce the precision of boundary delineation for very small muscles, which could affect the reliability of net CSA quantification. However, as the same protocol was applied uniformly to all participants, any measurement error is likely systematic and non‑differential, and thus unlikely to fully account for the observed between‑group differences. Previous anatomical studies (e.g., Xu et al., 2017) have employed comparable slice thicknesses [52]. Future prospective studies should utilize isotropic 3D sequences (slice thickness ≤ 1 mm) to improve measurement accuracy.

Second, **retrospective design.** The retrospective observational design precludes causal inference. While an association between RCPmi hypertrophy and PD was observed, it remains unclear whether this morphological feature contributes to disease pathogenesis or represents a secondary adaptive change. The retrospective data collection also resulted in missing clinical information (e.g., UPDRS scores), limiting the depth of confounding factor control.

Third, **lack of functional evidence.** This study did not perform quantitative measurements of CSF flow (e.g., phase-contrast MRI); therefore, the inference that “RCPmi hypertrophy affects CSF dynamics” remains speculative. A direct association between OAC morphological changes and CSF flow parameters has yet to be established.

Fourth, **lack of longitudinal data.** The cross-sectional design precludes tracking the dynamic evolution of RCPmi morphology over time and assessing the temporal relationship between RCPmi hypertrophy and disease progression.

Fifth, **limited sample size for subgroup analysis.** The correlation analysis for disease duration was limited to 51 patients with complete records. Although this subgroup showed no significant differences from the overall cohort in age, sex, or RCPmi net area, the relatively small sample size may affect statistical power and the generalizability of the findings.

Sixth, **selection bias.** Disease onset data collected via telephone follow-up may be subject to recall bias. Although healthy controls were screened to exclude cervical spondylosis and neck pain, other unidentified confounding factors may remain. PD patients were recruited from a single medical center, potentially introducing selection bias. Moreover, MDS-UPDRS III motor scores were completely unavailable for this retrospective cohort. Because no alternative measure of motor severity could be reliably derived from the existing data, we could not adjust for or analyze the potential contribution of axial motor symptoms to RCPmi hypertrophy. Consequently, we cannot exclude the possibility that the observed muscle changes are partially secondary to motor features such as axial rigidity. Additionally, although bilateral measurements were averaged for all analyses, formal statistical testing of side-to-side differences was not performed because the original left and right individual values were not retained in the final dataset. Consequently, we cannot completely rule out the possibility of undetected asymmetry in muscle CSA between the two sides. Future prospective studies should systematically evaluate and report side-to-side differences. Future prospective studies should systematically collect UPDRS-III scores to address this limitation.

### Clinical relevance

The observed association between RCPmi hypertrophy and disease duration suggests that RCPmi CSA could serve as a cross‑sectional imaging marker of disease chronicity in PD. However, several important limitations currently preclude its clinical application: (1) the need for validation in prospective cohorts with longitudinal follow‑up; (2) the inability to distinguish primary from secondary hypertrophy; (3) the absence of direct CSF flow measurements; and (4) the requirement for standardized imaging protocols (e.g., isotropic 3D sequences) to improve measurement precision. Therefore, while RCPmi morphology provides valuable research insights, its routine clinical use is not yet justified.

### Future directions

Future studies should address these limitations through the following approaches:

1. **Technical optimization**: Employ prospective imaging with isotropic 3D sequences to improve anatomical resolution and measurement accuracy; develop semi-automated or automated segmentation algorithms to enhance measurement reproducibility.
2. **Functional integration**: Integrate phase-contrast MRI to quantitatively assess CSF flow dynamics (velocity, volume, pulsatility) in the suboccipital region and examine the association between OAC morphological changes and CSF flow parameters.
3. **Longitudinal design**: Conduct longitudinal cohort studies to track dynamic changes in the RCPmi and surrounding structures over time and evaluate their temporal relationship with the progression of motor and non-motor symptoms in PD, clarifying their value as imaging biomarkers.
4. **Multi-center validation**: Perform multi-center studies with larger sample sizes to validate the reproducibility and generalizability of the current findings.
5. **Mechanistic exploration**: Integrate glymphatic function imaging techniques such as DTI-ALPS to directly assess the relationship between RCPmi morphology and glymphatic system function; explore targeted interventions (e.g., physical therapy, botulinum toxin injection) aimed at modulating RCPmi tone and evaluate their effects on CSF dynamics and clinical outcomes in PD.
6. **Clinical correlation**: Systematically collect clinical data, including UPDRS scores, cognitive assessments, and non-motor symptom scales, to conduct in-depth analyses of the associations between RCPmi morphological changes and clinical phenotypes.

## Conclusion

This study is the first to apply a standardized CSA ratio (muscle CSA / atlas posterior arch CSA) for morphological assessment of MDBC-related muscles in a Parkinson’s disease cohort. MRI evidence demonstrates selective hypertrophy of the RCPmi in PD patients, with the degree of hypertrophy positively correlated with both disease duration and OAC volume. Concurrent measurements of neural tissue anteroposterior diameters at the same anatomical levels as the OAC parameters revealed no significant differences between PD patients and HC subjects, suggesting that mechanical traction may be the primary mechanism underlying OAC enlargement. Based on the anatomical role of the RCPmi as a core component of the MDBC, we propose the following working hypothesis: pathological hypertrophy of the RCPmi may be associated with mechanical changes in the local CSF dynamic environment, which could theoretically interfere with the clearance efficiency of the GS for neurotoxic metabolites, but this requires functional validation. This hypothesis requires validation through further functional imaging and longitudinal studies. Morphological changes in the RCPmi hold promise as a potential imaging biomarker for monitoring disease progression in PD.

## Data Availability

The raw data supporting the fndings of this study are available from the corresponding authors upon reasonable request.

## Abbreviations

PD: Parkinson’s disease
CSA: Cross-sectional area
CSF: Cerebrospinal fluid
MDBC: Myodural bridge complex
GS: Glymphatic system
MDB: Myodural bridge
MRI: Magnetic resonance imaging
HC: Healthy controls
ICC: Intraclass correlation coefficient
RCPmi: Rectus capitis posterior minor
RCPma: Rectus capitis posterior major
OAC: Occipito-atlantal cistern
OCI: Obliquus capitis inferior
OCS: Obliquus capitis superior
RCL: Rectus capitis lateralis
SDM: Spinal dura mater
MO: Medulla oblongata
SC: Spinal cord
OCCI: Occipital bone and foramen magnum
C1: First cervical vertebra (atlas)
C2: Second cervical vertebra (axis)
TOC: Tonsils of the cerebellum
D-FM: Anteroposterior dimension of the dorsal subarachnoid space at the foramen magnum level
D-C1: Anteroposterior dimension of the dorsal subarachnoid space at the C1 (atlas) level
D-C2: Anteroposterior dimension of the dorsal subarachnoid space at the C2 (axis) level
AP: Anteroposterior
OC2A: Occiput-C2 angle
